# Tuning the Selectivity of Metal Oxide Gas Sensors with Vapor Phase Deposited Ultrathin Polymer Thin Films

**DOI:** 10.3390/polym15030524

**Published:** 2023-01-19

**Authors:** Stefan Schröder, Nicolai Ababii, Mihai Brînză, Nicolae Magariu, Lukas Zimoch, Mani Teja Bodduluri, Thomas Strunskus, Rainer Adelung, Franz Faupel, Oleg Lupan

**Affiliations:** 1 Multicomponent Materials, Department of Materials Science, Faculty of Engineering, Kiel University, Kaiserstraße 2, D-24143 Kiel, Germany; 2 Center for Nanotechnology and Nanosensors, Department of Microelectronics and Biomedical Engineering, Technical University of Moldova, 168 Stefan cel Mare Av., MD-2004 Chisinau, Moldova; 3 Functional Nanomaterials, Department of Materials Science, Faculty of Engineering, Kiel University, Kaiserstraße 2, D-24143 Kiel, Germany; 4 Fraunhofer Institute for Silicon Technology (ISIT), Fraunhoferstraße 1, D-25524 Itzehoe, Germany

**Keywords:** gas sensors, PV4D4 polymer, thin films, hybrid materials, 2-propanol, selectivity, initiated chemical vapor deposition

## Abstract

Metal oxide gas sensors are of great interest for applications ranging from lambda sensors to early hazard detection in explosive media and leakage detection due to their superior properties with regard to sensitivity and lifetime, as well as their low cost and portability. However, the influence of ambient gases on the gas response, energy consumption and selectivity still needs to be improved and they are thus the subject of intensive research. In this work, a simple approach is presented to modify and increase the selectivity of gas sensing structures with an ultrathin polymer thin film. The different gas sensing surfaces, CuO, Al_2_O_3_/CuO and TiO_2_ are coated with a conformal < 30 nm Poly(1,3,5,7-tetramethyl-tetravinyl cyclotetrasiloxane) (PV4D4) thin film via solvent-free initiated chemical vapor deposition (iCVD). The obtained structures demonstrate a change in selectivity from ethanol vapor to 2-propanol vapor and an increase in selectivity compared to other vapors of volatile organic compounds. In the case of TiO_2_ structures coated with a PV4D4 thin film, the increase in selectivity to 2-propanol vapors is observed even at relatively low operating temperatures, starting from >200 °C. The present study demonstrates possibilities for improving the properties of metal oxide gas sensors, which is very important in applications in fields such as medicine, security and food safety.

## 1. Introduction

Metal oxide gas sensors are developing very rapidly and extensively, due to their use in different fields, ranging from natural sciences to nanobiomedicine, space innovations, the oil and food industries and the automotive industry [1,2]. The reasons are their superior properties such as response and selectivity to different gases and volatile organic compounds (VOCs) [3,4,5,6], portability and selective sensitivity adjustment combined with ultra-low power consumption [7,8] and the nanoscale device dimensions [9,10]. However, energy consumption and gas selectivity of metal oxide gas sensors could be further improved and are thus of great interest in current research. Furthermore, the operation of this sensor type in ambient conditions, especially at high humidity levels is hindered by the moisture and environment, since overlap interference with various gas species is still a challenge for them [11,12]. Conductive polymers as emerging layers have gained increasing attention for sensor application due to their unique properties, low cost and processing procedure [13]. Beniwal et al. developed SnO_2_/polypyrrole (PPy) nanocomposite by electrospinning approach for 100 ppb NH_3_ sensing device structure obtaining a response of 57% [14]. PPy possesses a secondary amine group (-NH-) which interacts with NH_3_ vapor and thus increases the response. Furthermore, Zhang et al. synthesized PPy/Zn_2_SnO_4_ nanolayer for room temperature NH_3_ gas sensor [15]. In addition, polyaniline (PANI) was found as a polymer material with potential gas sensing applications. Sáaedi et al. developed ZnO/PANI nanocomposite as gas sensing films, which lower the operating temperature and increases the response of the device approximately four times compared to the original ZnO thin film device when exposed to methanol vapor [16]. Another recently investigated approach is based on the modification of conducting polymers by involving metal nanoparticles, nano-metal oxides, and carbon nanomaterials to improve the response/sensitivity, stability and reproducibility of the overall sensor characteristics [17]. Modern developments in synthesis approaches have facilitated the fabrication of various types of polymer sensors for a wide range of applications, starting from bioengineering/biomedical, smart clothes and energy harvesting up to modern robotics, etc [18]. We recently demonstrated the improved performance of CuO/Cu_2_O/ZnO:Fe gas sensing heterostructures covered with ultrathin 25 nm hydrophobic cyclotrisiloxane-based polymer thin films in high humidity environments [19]. The deposition of these tailored thin films was enabled by solvent-free initiated chemical vapor deposition (iCVD) using 1,3,5-trimethyl-trivinyl cyclotrisiloxane (V3D3) monomer combined with perfluorobutanesulfonyl fluoride (PFBSF) initiator. This polymer thin film provides a hydrophobic coating and is in addition stable at high operating temperatures required for the metal oxide gas sensing demonstrated for several measurement cycles and humidity-independent sensor structure. Another benefit associated with the additional ultrathin polymer V3D3 coating is a change in sensor selectivity towards hydrogen gas [19].

In this study, we investigate whether we can further tune/control the selectivity of gas sensors by replacing the V3D3 monomer with 1,3,5,7-tetramethyl-tetravinyl cyclotetrasiloxane (V4D4) monomer in the iCVD process. PV4D4 was chosen because it has similar hydrophobic properties as PV3D3 but a larger ring structure, which could shift the specific selectivity to larger molecules such as 2-propanol. Three different gas sensing structures, copper oxide (CuO), aluminum oxide/copper oxide heterostructures (Al_2_O_3_/CuO) and titanium dioxide (TiO_2_), are each coated with a nanoscale PV4D4 thin film via iCVD. The responses to n-butanol, 2-propanol, ethanol and acetone are measured and compared to the respective responses of uncoated structures without PV4D4 thin films. The iCVD process is a solvent-free polymer thin film deposition process from the vapor phase [20,21]. The underlying reaction is a free radical polymerization at a substrate cooled to room temperature. Due to its CVD-typical growth characteristics, it can be easily scaled up and applied to coat complex geometries such as porous substrates or gas sensing structures as described in this study. For the deposition initiator and monomer vapors are introduced to a hot filament reactor. After the filaments are heated, the initiator is decomposed into free radicals, which start polymerization with the monomers adsorbed at the cooled substrate stage. The sizes of the cyclotetrasiloxane rings of the respective PV4D4 molecules are calculated by geometry optimization via density functional theory (DFT). It can help to explain and support the detection mechanism.

## 2. Materials and Methods

**Figure 1:**
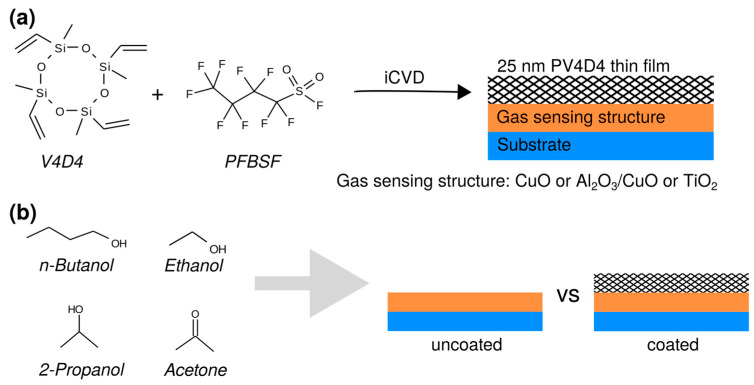
Schematic illustration of the study: (**a**) CuO, Al_2_O_3_/CuO and TiO_2_ gas sensors are each coated with a 25 nm PV4D4 thin film grown from V4D4 monomer and PFBSF initiator using the iCVD process; (**b**) the coated and uncoated gas sensors are then exposed to different volatile organic vapors and compared based on their individual gas responses.

We investigate three different gas sensing structures, CuO, Al_2_O_3_/CuO and TiO_2_ coated with a 25 nm PV4D4 thin film via iCVD by using a V4D4 monomer and PFBSF initiator, illustrated in Figure 1a. We test their response to various volatile organic compounds, namely n-butanol, 2-propanol, ethanol and acetone, and compare the respective sensor responses to uncoated structures without PV4D4 thin films. The structural formulas of these four vapors are shown in Figure 1b.

### 2.1. Sensor Fabrication

In order to obtain the sensor structures described in this study, several methods were used. The CuO structures were obtained by sputtering metallic copper under vacuum conditions onto glass substrates at 25 °C using a customized RF-magnetron system. Then, the structures were heat treated under normal atmospheric conditions according to a previous work [22]. For the Al_2_O_3_/CuO structures, a combination of two methods was used. The CuO films were obtained by the synthesis of chemical solutions (SCS approach) on a glass substrate [23,24] followed by a heat treatment in different regimes. Subsequently, an ultrathin film of Al_2_O_3_ was deposited by atomic layer deposition (ALD) at a deposition temperature of 75 °C. In the end, the samples were annealed according to the processes described in a previous work [25]. The TiO_2_ structures were, like the Al_2_O_3_ films, obtained by ALD on a glass or ceramic substrate. Additional details on the deposition parameters are described in a previous work [26].

After the fabrication of the three different gas sensing structures, a 25 nm PV4D4 thin film was deposited on each of the structures in a custom-made iCVD setup reported elsewhere [27,28,29]. A rotary vane pump (Pfeiffer Vacuum Duo 10) was used to evacuate the reactor. The monomer V4D4 (Abcr, 97%) and initiator PFBSF (Chempur, 95%) were delivered via low-flow metering valves at flow rates of 0.2 sccm and 0.1 sccm, respectively, to the reactor. The setup was handled in continuous flow mode and a process pressure of 40 Pa was maintained by a butterfly valve (VAT 615) coupled to a capacitive manometer (MKS Baratron). The reactor and all monomer and exhaust lines were heated to 110 °C to prevent monomer condensation. The substrate stage was cooled by a thermostat (Huber CC-K6) to 32 °C. A filament array consisting of NiCr wire (Goodfellow) is located 30 mm above the substrate stage and was resistively heated using 64.05 W during the deposition with a power supply.

### 2.2. Sample Characterization

The morphology of the CuO, Al_2_O_3_/CuO and TiO_2_ uncoated and PV4D4-coated gas sensor structures were investigated using scanning electron microscopy (SEM, Carl Zeiss). The acceleration voltage was set to 3 kV and 7 kV for PV4D4-coated and uncoated structures, respectively. The gas sensing measurements for the coated and uncoated CuO, Al_2_O_3_/CuO and TiO_2_ structures were performed in a special custom-made chamber according to a protocol reported in a previous study [30]. All samples were inserted, electrically connected and heated to the desired working temperature for 45 min before applying the test gases. In order to obtain the gas response in % scale, the formula

(1) $$S=\frac{G_{gas}-G_{air}}{G_{air}}\times 100\%$$

was used [31,32]. S is the response in %, *G_gas_* is the electrical conductance of the specimen in gas and *G_air_* represents the conductance of the same specimen in air. FTIR measurements were performed using an FTIR spectrometer (Bruker Invenio-R). 32 scans were performed from 400 cm^−1^ to 4000 cm^−1^ with a resolution width set to 4 cm^−1^. The measured spectra were baseline corrected using a scientific graphing program (Origin 2017). Geometry optimization of the V4D4 molecule was performed using density functional theory (DFT). For this purpose, the hybrid functional B3LYP [33,34,35] was applied together with the cc-pVDZ basis [36] set in the program NWChem [37].

## 3. Results and Discussion

### 3.1. Morphological Characterization

**Figure 2:**
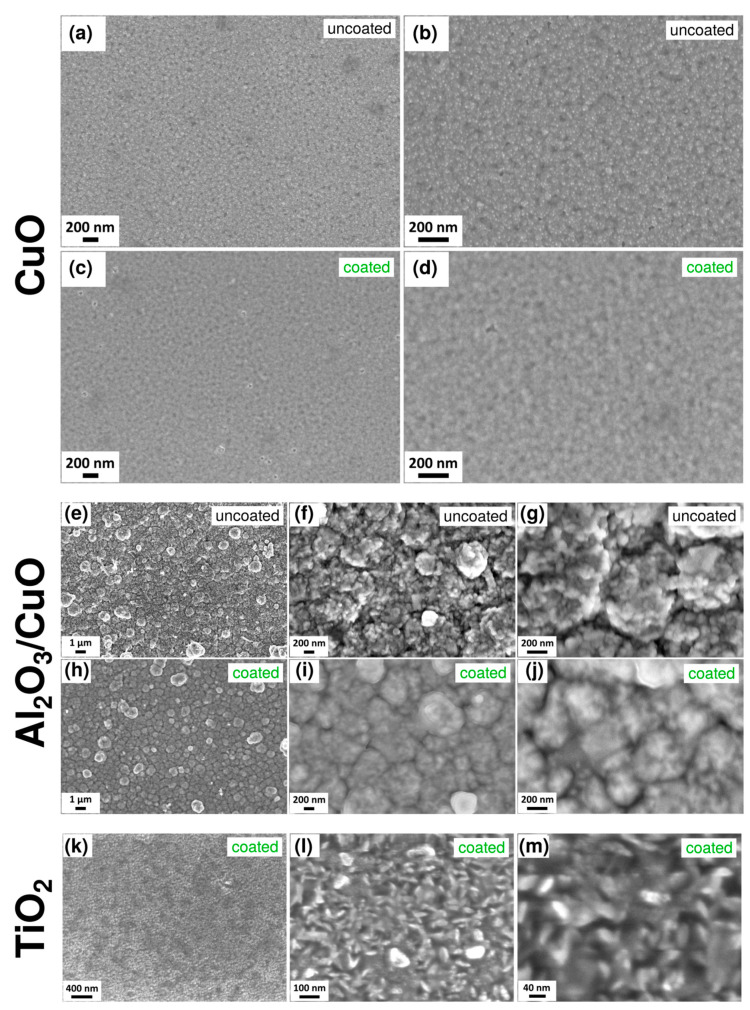
SEM images of the morphologies of the three different gas sensor structures investigated in this study. Uncoated CuO structure grown by sputtering at: (**a**) low magnification and (**b**) high magnification. SEM images of the CuO structure grown by sputtering then covered with PV4D4 thin film at (**c**) low magnification and (**d**) high magnification. Uncoated Al_2_O_3_/CuO obtained by ALD/SCS approaches and measured at magnification (**e**) 1 µm; (**f**) and (**g**) 200 nm scale bar. SEM images of the Al_2_O_3_/CuO by ALD/SCS covered on top with PV4D4 thin film at (**h**) 1 µm; (**i**) and (**j**) 200 nm scale bar. TiO_2_ structures with a thickness of 30 nm and a 25 nm PV4D4 thin film on top measured at (**k**) low magnification; (**l**) medium magnification; and (**m**) high magnification by SEM.

**Figure A1:**
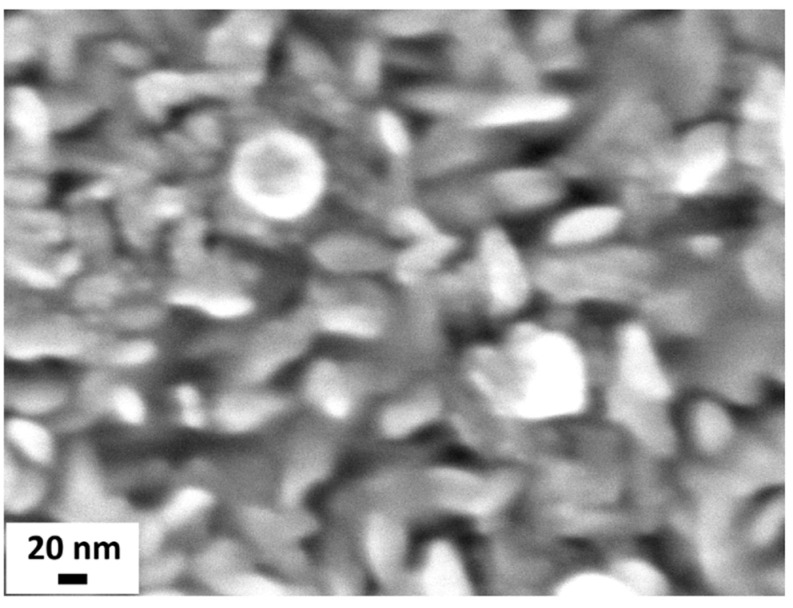
SEM image of the TiO_2_ structure with 25 nm thickness without PV4D4 polymer thin film.

In this section, the surface morphology of the structures and heterostructures is presented. Due to the CVD-typical synthesis characteristics of iCVD complex geometries, such as the gas sensing structures in this study, can typically be homogeneously coated. Figure 2a–d shows the SEM images of the uncoated CuO surface structure compared to a CuO structure coated with a 25 nm PV4D4 thin film. Figure 2a,b reveals that the CuO film consists of penetrating nanogranules that completely cover the glass substrate. After the deposition of the nanometric PV4D4 thin film (Figure 2c,d), a smoothing of the nanogranules can be observed due to uniform polymer deposition. In Figure 2e–j, the SEM images of the second type of gas sensor, Al_2_O_3_/CuO, investigated in this study are shown. The SEM images show the structure of uncoated (Figure 2e–g) and PV4D4-coated structures (Figure 2h–j) at different magnifications. After deposition of the PV4D4 coating, a smoothening of the surface can again be observed, which indicates that the polymer was deposited conformally on the entire surface of the structure. Figure 2k–m shows SEM images of the third type of gas sensing structure, TiO_2_. The thickness of the TiO_2_ in the SEM-investigated samples is 30 nm. Different magnifications of the PV4D4-coated TiO_2_ are shown. The structure covers the glass substrate completely and continuously (Figure 2k). Figure 2k,l reveals that the homogeneous granular surface and the grains correspond to small aggregates of nano- and microcrystallites. For comparison, Figure A1 in the appendix shows the SEM image of the TiO_2_ structure without the PV4D4 thin film.

### 3.2. Chemical Characterization

**Figure 3:**
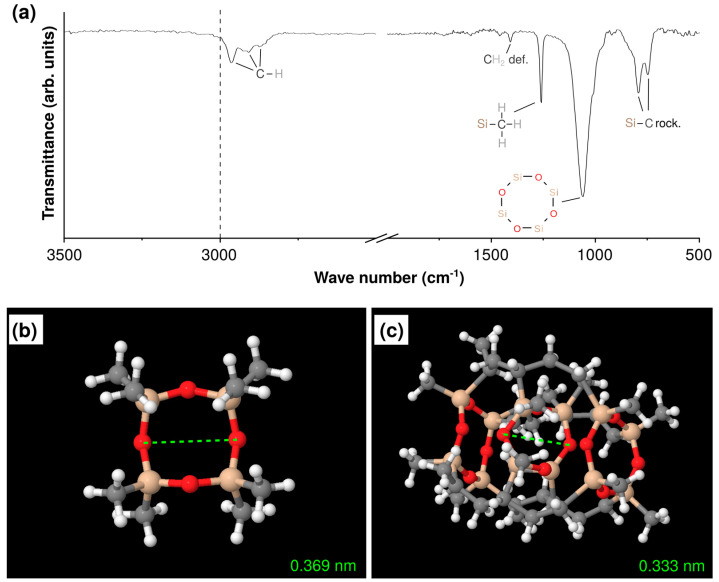
Chemical characterization and theoretical estimation of PV4D4 ring structures. (**a**) The infrared bands measured for the deposited PV4D4 thin films via FTIR show preserved functionality of all functional groups as well as successful polymerization; (**b**) isolated D4 molecule geometry optimized using DFT on the B3LYP/6-31G* level; and (**c**) embedded V4D4 molecule connected at all sites only to one other V4D4 molecule geometry optimized via DFT.

For the chemical characterization of the deposited PV4D4 thin films FTIR measurements have been performed. The obtained spectra are shown in Figure 3a. The absence of vinyl groups in the spectrum indicates that the deposited PV4D4 thin film has been successfully polymerized. Vinyl groups are present in the V4D4 monomer and should give rise to a band >3000 cm^−1^ for the C-H stretch at a vinyl group [38]. Furthermore, the clearly visible band at 1057 cm^−1^ reveals, that the cyclotetrasiloxane ring was preserved during the polymerization [39]. The ring structure was not decomposed during the deposition and shows full functionality. In order to obtain information on the size of the cyclotetrasiloxane ring geometry optimizations of the respective molecule are performed using density functional theory (DFT) on the B3LYP/cc-pVDZ level. Two situations are investigated to estimate the maximum and minimum diameter of the ring. The first calculation is performed at an isolated D4 molecule, shown in Figure 3b, and the second at a V4D4 molecule, which is bonded at each site only to one other V4D4 molecule (Figure 3c). For the isolated octamethylcyclotetrasiloxane (D4) molecule, the smallest distance is found to be 0.369 nm across the two opposite oxygen atoms. The embedded molecule represents the smallest situation resulting from free radical polymerization, which is the underlying reaction in iCVD [40,41]. The smallest diameter in the embedded cyclotetrasiloxane ring is 0.333 nm measured between the two opposite oxygen atoms. As each molecule of the four investigated vapors is larger than the diameter of the cyclotetrasiloxane ring, it can be assumed that the transport of the vapor molecules through the PV4D4 film to the sensor surface might take place only via the remaining free volume of the polymer and not through the cyclotetrasiloxane rings. However, the more open structure of PV4D4 compared to PV3D3 should also give rise to more available free volume.

### 3.3. Gas Sensing Properties

**Figure 4:**
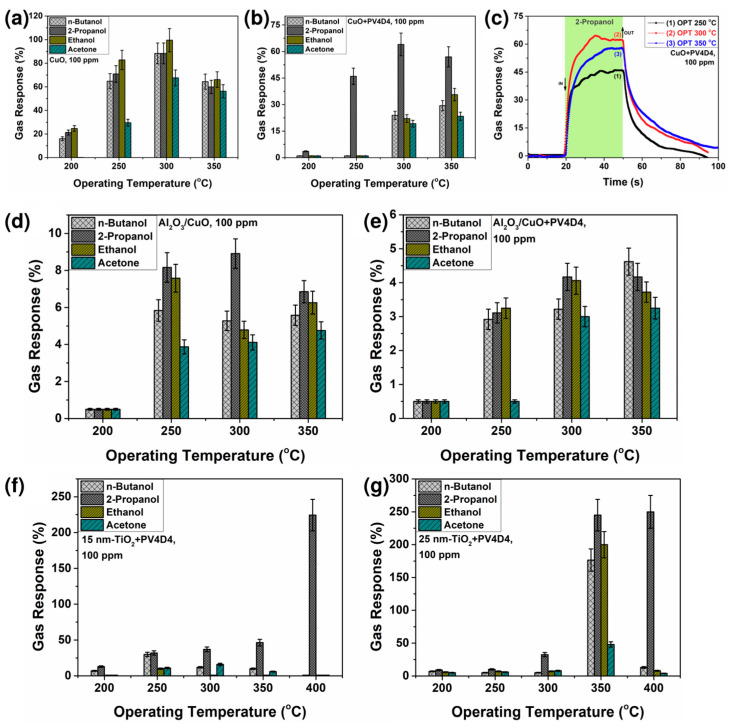
Gas response versus operating temperature for the (**a**) uncoated CuO structures, grown by sputtering; and (**b**) CuO structures, grown by sputtering and then coated with PV4D4 thin film. (**c**) Dynamic responses to 100 ppm of 2-propanol for the CuO structures with PV4D4 thin film. Gas response versus operating temperature for the (**d**) uncoated Al_2_O_3_/CuO structures, grown by ALD/SCS; and (**e**) Al_2_O_3_/CuO structures, grown by ALD/SCS, then coated with PV4D4 thin film on top. Gas response versus operating temperature for the TiO_2_ structures, grown by ALD, then coated with PV4D4 thin film. The investigated TiO_2_ structures are (**f**) 15 nm and (**g**) 25 nm thick.

**Figure A2:**
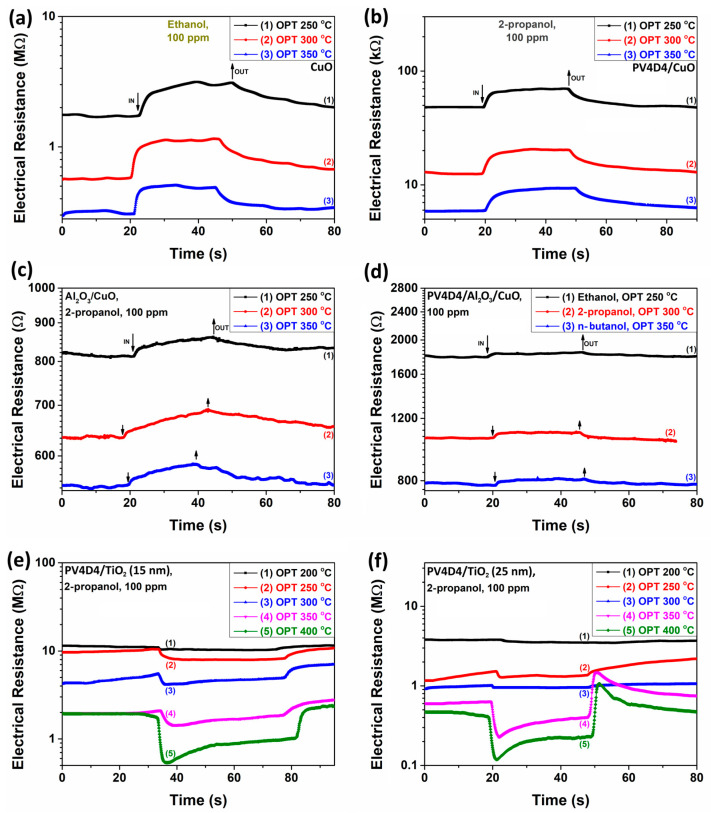
Electrical resistance at different operating temperature for the: (**a**) uncoated CuO structures to ethanol vapor; and (**b**) CuO structures with PV4D4 thin film to 2-propanol vapor. Electrical resistance at different operating temperature for the: (**c**) uncoated Al_2_O_3_/CuO structures to 2-propanol vapor; and (**d**) Al_2_O_3_/CuO structures with PV4D4 thin film to different vapor. Electrical resistance at different operating temperature for the TiO_2_ structures with PV4D4 thin film to 2-propanol vapor. The investigated TiO_2_ structures are: (**e**) 15 nm and (**f**) 25 nm thick.

In this section, the gas sensing properties of the three different structure types are described and the performance change attributed to the additional PV4D4 thin film is demonstrated. Additional electrical resistance measurements of all PV4D4/metal oxide gas sensing structures can be found in Figure A2 in Appendix A. Figure 4a shows the gas response of an uncoated CuO structure, grown by sputtering, for n-butanol, 2-propanol, ethanol and acetone with a concentration of 100 ppm versus working temperature. The measurement shows that at operating temperatures between 250 °C and 350 °C, the sensing structure responds to all vapors. The selectivity for ethanol vapors predominates, which is in accordance with the data from the literature [23,42]. The highest response to ethanol vapor has a value of ~100% at an operating temperature of 300 °C. The gas response of the CuO sensor coated with the PV4D4 thin film, presented in Figure 4b, shows a slightly different behavior. The coated structures were also exposed to n-butanol, 2-propanol, ethanol and acetone vapor with a concentration of 100 ppm and measured at different working temperatures. The measurement shows that the polymer deposited on top of the metal oxide sensor influences the gas response in a way that the selectivity changes to 2-propanol vapor with an optimum operating temperature of 300 °C. The responses to 2-propanol vapor are ~3.6%, ~46%, ~64% and ~57% at operating temperatures of 200 °C, 250 °C, 300 °C and 350 °C, respectively. Figure 4c shows the dynamic responses of the PV4D4-coated CuO structures to 100 ppm of 2-propanol. The measurement shows again an optimum operating temperature of 300 °C and all pulses return to their initial value after stopping the test gas, which indicates improved sensor performances of the developed structures.

The response/recovery times (defined as the necessary time to reach 90% of the full response value) were determined from the dynamic response measurement (Figure 4c). The values are presented in Table 1.

The gas response measurements for the second gas sensing structures, Al_2_O_3_/CuO, grown by ALD/SCS approaches, then coated with PV4D4 thin film on top are shown in Figure 4d,e. Figure 4d shows the response of the uncoated Al_2_O_3_/CuO structures to n-butanol, 2-propanol, ethanol and acetone vapor with a concentration of 100 ppm versus operating temperature. The structure responds to all vapors at working temperatures between 250 °C and 350 °C. The selectivity to 2-propanol vapor predominates. The 2-propanol response at the working temperatures of 250 °C, 300 °C and 350 °C have values of ~8%, ~8.9% and ~6.8%, respectively. Figure 4e results indicate that the additional PV4D4 coating shows here no significant influence on the responses to the vapors.

The third type of gas sensing structure investigated in this study are 15 nm and 25 nm TiO_2_ structures, grown by ALD, then coated with an additional PV4D4 thin film. Figure 4f shows the gas response of the TiO_2_ structures of 15 nm thickness coated with PV4D4 to n-butanol, 2-propanol, ethanol and acetone vapor with a concentration of 100 ppm versus working temperature. The measurement reveals that these structures are more selective to 2-propanol vapor at all working temperatures. At an operating temperature of 400 °C, the highest response with a value of 225% is obtained for such devices. TiO_2_ structures with a slightly larger thickness of 25 nm coated with PV4D4 show similar behavior, as shown in Figure 4g. Again, the response to n-butanol, 2-propanol, ethanol and acetone with a concentration of 100 ppm is investigated at different operating temperatures. The coated structures of the third type are selective for 2-propanol at all operating temperatures. It can also be observed that an increase in structure thickness results in the highest responses at working temperatures of 350 °C and 400 °C. The respective values are 245% and 250%. PV4D4 has a larger ring structure compared to PV3D3 used in our previous study [19]. PV4D4 seems to shift the specific selectivity to larger molecules such as 2-propanol. TiO_2_, coated with a 25 nm PV4D4 thin film via iCVD shows a clear performance for 2-propanol and indicates that the coverage with an additional polymer thin film can help to control the sensitivity and selectivity of metal oxide gas sensors.

To highlight the novelty of our work in comparison to previous works we provide a summary in Table 2. It shows a comparison of the sensors based on reported conducting polymer/metal oxide hybrids and the sensors based on the dielectric iCVD PV4D4/metal oxide hybrids developed and studied in this work.

## 4. Conclusions

In this work, we have reported on the surface modification of CuO, Al_2_O_3_/CuO and TiO_2_ gas sensing structures covered on top with a PV4D4 polymer nanoscale thin film deposited via solvent-free initiated chemical vapor deposition. FTIR measurements have been performed for the chemical characterization of the deposited PV4D4 thin films. The absence of vinyl groups in the spectrum indicated that these layers have been successfully polymerized and the ring structure was not decomposed during the deposition showing complete functionality. The size of the cyclotetrasiloxane ring geometry optimizations of the respective molecule was performed using DFT on the B3LYP/cc-pVDZ level. The embedded molecule showed the smallest distance when combined in the polymerization. The gas responses of the coated and uncoated gas sensors were tested for four volatile organic compounds, n-butanol, 2-propanol, ethanol and acetone. The presented approach enables to change the selectivity of CuO gas sensing structures from ethanol vapor to 2-propanol vapor. The highest response, ~64%, was observed at 300 °C working temperature. Al_2_O_3_/CuO gas sensing structures show no significant change in gas response when coated with the additional PV4D4 thin film. In the case of PV4D4-coated TiO_2_ structures with different thicknesses (15 nm and 25 nm), the selectivity for 2-propanol vapor also increases even at relatively low operating temperatures. At working temperatures of 400 °C, a gas response of 225% was obtained for PV4D4/TiO_2_ structures with a TiO_2_ structure thickness of 15 nm and responses of 245% and 250% were obtained at working temperatures of 350 °C and 400 °C for the respective PV4D4/TiO_2_ structure with 25 nm thickness. The main observation is that PV4D4/TiO_2_ coated structures are selective for 2-propanol at all operating temperatures. Geometry optimization reveals, that the transport might take place exclusively through the remaining free volume and not via the cyclotetrasiloxane rings in the polymer film. The obtained results are very promising in the field of metal oxide-based gas sensors since a simple approach can be used to change and increase the selectivity from one type of vapor to another and to protect its surface in various ambient conditions.

## Figures and Tables

**Table 1:** Response and recovery times of CuO structures with PV4D4 coating to 2-propanol vapor.

Operating Temperature (°C)	Response Time (s)	Recovery Time (s)
250	8.6	19.6
300	9.5	35
350	11.4	40

**Table 2:** Comparison of sensors based on conducting polymer/metal oxide hybrids and gas sensors based on the dielectric iCVD PV4D4/metal oxide hybrids studied in this work.

Sensor Material	Polymer	Analyte	Response	Working Temp (°C)
SnO_2_ [14]	(PPy) Polypyrrole	NH_3_	57% (0.1 ppm)	RT
ZnO [43]	PT nanofibers	NO	22.8% (308 ppm)	RT
MnO_2_ [44]	(PPy) Polypyrrole	NH_3_	46.44 (5 ppm)	RT
ZnO [16]	(PANi) Polyaniline	Methanol	19.2 (100 ppm)	60
Fe_2_O3 [45]	(PANi) Polyaniline	NH_3_	3.79 (100 ppm)	RT
WO_3_ [46]	Polythiophene	H_2_S	13 (100 ppm)	70
Boron nitride-Pt nanoparticles [47]	(PANi) Polyaniline	Glucose	19.2 mAM^−1^cm^−2^	60
TiO_2_ nanotube-Au nanoparticles [48]	(PANi) Polyaniline	Lactate	0.0401 μAμM^−1^cm^−2^	
ZnO [49]	(PPy) Polypyrrole	Xanthine	N.A.	35
Cu_x_O [50]	(PPy) Polypyrrole	Glucose	232.22 μAmM^−1^cm^−2^	RT
Fe_3_O_4_ [51]	(PPy) Polypyrrole	Glucose	-	RT
TiO_2_-GO_x_ [52]	(PANi) Polyaniline	Glucose	6.31 μAmM^−1^cm^−2^	
Graphene [53]	(PEDOT) 3, 4-ethylenedioxythiophene	Ascorbic acid	-	RT
Graphene [54]	(PEDOT) 3, 4-ethylenedioxythiophene	Dopamine	-	RT
**CuO (this work)**	PV4D4	2-propanol	70%(100 ppm)	300
**Al_2_O_3_/CuO (this work)**	PV4D4	n-butanol	4.5%(100 ppm)	350
**TiO_2_ (this work)**	PV4D4	2-propanol	225%(100 ppm)	400
**TiO_2_ (this work)**	PV4D4	2-propanol	251%(100 ppm)	350

## Data Availability

Not applicable.

## Appendix A

Additional measurements for selected samples are shown here in the appendix.
